# Development of a life expectancy table for individuals with type 1 diabetes

**DOI:** 10.1007/s00125-021-05503-6

**Published:** 2021-07-26

**Authors:** An Tran-Duy, Josh Knight, Philip M. Clarke, Ann-Marie Svensson, Björn Eliasson, Andrew J. Palmer

**Affiliations:** 1 Centre for Health Policy, Melbourne School of Population and Global Health, The University of Melbourne, Melbourne, VIC Australia; 2 Health Economics Research Centre, Nuffield Department of Population Health, University of Oxford, Old Road Campus, Headington, UK; 3 Department of Molecular and Clinical Medicine, Institute of Medicine, University of Gothenburg, Gothenburg, Sweden; 4 Swedish National Diabetes Register, Centre of Registers, Gothenburg, Sweden; 5 Menzies Institute for Medical Research, The University of Tasmania, Hobart, Tasmania Australia

**Keywords:** Clinical decision support, Computer simulation, Decision aids, Education, Life expectancy, Mathematical modelling, Mortality, Risk factors, Type 1 diabetes, Visual tools

## Abstract

**Aims/hypothesis:**

Tables reporting life expectancies by common risk factors are available for individuals with type 2 diabetes; however, there is currently no published equivalent for individuals with type 1 diabetes. We aimed to develop a life expectancy table using a recently published simulation model for individuals with type 1 diabetes.

**Methods:**

The simulation model was developed using data from a real-world population of patients with type 1 diabetes selected from the Swedish National Diabetes Register. The following six important risk factors were included in the life table: sex; age; current smoking status; BMI; eGFR; and HbA_1c_. For each of 1024 cells in the life expectancy table, a synthetic cohort containing 1000 individuals was created, with other risk factors assigned values representative of the real-world population. The simulations were executed for all synthetic cohorts and life expectancy for each cell was calculated as mean survival time of the individuals in the respective cohort.

**Results:**

There was a substantial variation in life expectancy across patients with different risk factor levels. Life expectancy of 20-year-old men varied from 29.3 years to 50.6 years, constituting a gap of 21.3 years between those with worst and best risk factor levels. In 20-year-old women, this gap was 18.9 years (life expectancy range 35.0–53.9 years). The variation in life expectancy was a function of the combination of risk factor values, with HbA_1c_ and eGFR consistently showing a negative and positive correlation, respectively, with life expectancy at any level combination of other risk factors. Individuals with the lowest level (20 kg/m^2^) and highest level of BMI (35 kg/m^2^) had a lower life expectancy compared with those with a BMI of 25 kg/m^2^. Non-smokers and women had a higher life expectancy than smokers and men, respectively, with the difference in life expectancy ranging from 0.4 years to 2.7 years between non-smokers and smokers, and from 1.9 years to 5.9 years between women and men, depending on levels of other risk factors.

**Conclusions/interpretation:**

The life expectancy table generated in this study shows a substantial variation in life expectancy across individuals with different modifiable risk factors. The table allows for rapid communications of risk in an easily understood format between healthcare professionals, health economists, researchers, policy makers and patients. Particularly, it supports clinicians in their discussion with patients about the benefits of improving risk factors.

**Graphical abstract:**

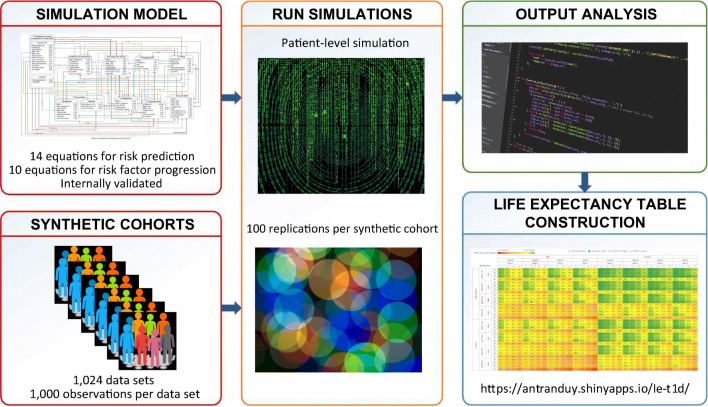

**Supplementary Information:**

The online version contains peer-reviewed but unedited supplementary material available at 10.1007/s00125-021-05503-6.

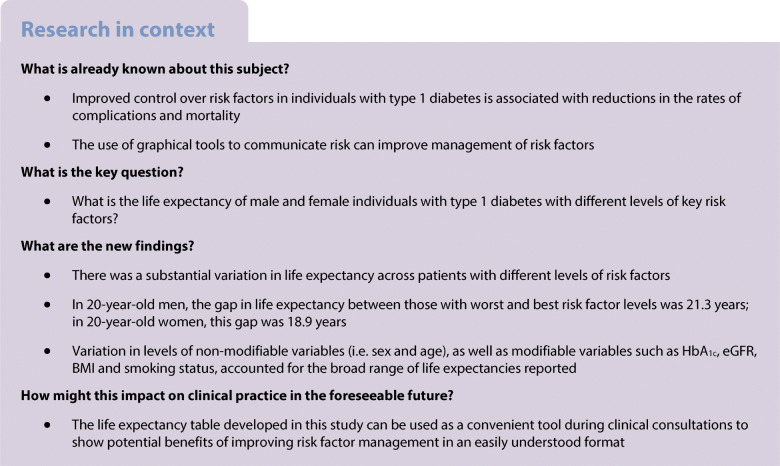



## Introduction

Despite increasing gains in life expectancy, individuals with type 1 diabetes have a life expectancy 10–12 years lower than the general population [1, 2]. While the gap had been reduced over the second half of the twentieth century [3], the evidence on the continuation of this improvement in the twenty-first century is mixed [1, 4]. Optimal control over risk factors has been shown to reduce the occurrence of diabetes-related adverse events [5–7] and all-cause mortality [7, 8]. The delivery of personalised risk information for patients has been shown to be effective at improving the management of modifiable risk factors in individuals with chronic diseases [9] and likely improving patients’ acceptance of doctor-recommended management strategies [10, 11].

Currently, risk information predominantly relates to the risk of CVD over a 5 or 10 year period [12, 13], and includes both risk charts [14] and risk calculators [15]. While such risk charts assist in prescribing of medications [16], patients may also value information on a broader range of health outcome measures including life expectancy. Recent reviews suggest the importance of discussions on prognosis of patients with serious health conditions [17, 18]. For patients with type 2 diabetes, life expectancies stratified by combinations of risk factor levels have been produced [19].

Decision aids that support patients by making their decisions explicit and providing more information about options and benefits/harms have been shown to increase patient preferences for effective CVD risk-reducing strategies and increase the number of patients choosing to start new medications for diabetes [20]. The use of visual tools such as colour-coded charts has been shown to be associated with higher levels of patient understanding compared with verbal and numerical (e.g. absolute or relative percentages, frequencies) presentations [21]. The expression of risk-related concepts in natural units such as life expectancy is easy for patients to interpret and has a higher level of recall compared with the relative measure of risk [22].

There is evidence that neither type 2 diabetes-specific nor general CVD risk algorithms adequately predict CVD risk in individuals with type 1 diabetes [23, 24], and there are currently no life expectancy tables for these patients. The recent development of a comprehensive simulation model that was based on a series of type 1 diabetes-specific equations enables the estimation of a range of end points, including risks of CVD, amputation, hypoglycaemia, hyperglycaemia and death [25]. This model is characterised by the multifactorial nature of type 1 diabetes progression, a wide range of interdependent endpoints, and complex forms of the risk equations [25]. While the direct use of such a complex model to discuss risks with patients is impractical, it can be used to estimate life expectancy of patients with pre-specified risk factors and therefore enables the development of a life expectancy table. The availability of life expectancy estimates stratified by combinations of risk factor levels allows for efficient communications of the impact of modifications of important risk factors in units that are understandable to patients. Such life expectancy tables facilitate an immediate assessment of the capacity to improve health outcomes by improving modifiable risk factors [26]. Given the lack of a visual tool for risk communication in type 1 diabetes, we aimed to develop a risk factor-based life expectancy table based on a simulation model derived from a nationwide population of patients with type 1 diabetes [25, 27].

## Methods

### Study design: principles and rationale

We developed a life expectancy table for individuals with type 1 diabetes by running simulations using our recently published model to predict survival times of individuals with specific baseline characteristics [25]. Variables required as input for the simulations included 25 risk factors (see Table 1) and 16 other auxiliary variables such as time since last hyperglycaemia in patients with a history of this event (see Electronic supplementary material [ESM] Table 1). As the number of the input variables was large, we balanced the number of risk factors to be included in the life expectancy table with the table size to facilitate its use in practice. Therefore, we selected six of the most important risk factors for the table (including sex, age, current smoking status, BMI, eGFR and HbA_1c_). These had been identified in the sensitivity analyses in our previous study [25] as being risk factors for which changes in their values had a greater impact on life expectancy compared with other variables. For these risk factors, we selected two levels for the binary variables and four levels for the continuous variables as follows:

- Sex: male and female
- Current age, in years: 20, 30, 40 and 50
- Current smoking status: smoker and non-smoker
- Baseline BMI, in kg/m^2^: 20, 25, 30 and 35
- Baseline eGFR, in ml min^−1^ [1.73 m]^−2^: 30, 60, 90 and 120
- HbA_1c_, in mmol/mol (%): 42 (6), 64 (8), 86 (10) and 108 (12)

**Table 1:** Baseline characteristics of individuals with type 1 diabetes in the real-world population (*n* = 27,841) used to develop the synthetic cohorts

Characteristic	Real-world population baseline value	Used in the synthetic cohort and life expectancy table development
Male sex, *n* (%)	15,492 (55.6)	Separate cohorts for men and women; risk factor displayed in the life expectancy table
Current smoker, *n* (%)	3783 (13.6)	Separate cohorts for smokers and non-smokers; risk factor displayed in the life expectancy table
Age, years	36.98 ± 14.94	Mean value within the range from lowest level to highest level for each risk factor; risk factors displayed in the life expectancy table
BMI, kg/m^2^	24.90 ± 3.71	
eGFR, ml min^−1^ [1.73 m]^−2^	96.05 ± 26.52	
HbA_1c_, mmol/mol	65 ± 13	
HbA_1c_, %	8.10 ± 1.38	
Age at onset, years	15.01 ± 7.60	Mean values assigned to each individual in any synthetic cohort; risk factors hidden in the life expectancy table
Systolic BP, mmHg	127.30 ± 16.91	
Triacylglycerols, mmol/l	1.16 ± 0.85	
HDL-cholesterol, mmol/l	1.59 ± 0.46	
LDL-cholesterol, mmol/l	2.72 ± 0.82	
Former smoker, *n* (%)	1202 (4.3)	Joint distribution of the proportions used to sample the values of these risk factors; risk factors hidden in the life expectancy table
Microalbuminuria, *n* (%)	5848 (21.0)	
Macroalbuminuria, *n* (%)	2299 (8.3)	
History of MI, *n* (%)	802 (2.9)	
History of stroke, *n* (%)	488 (1.8)	
History of CHF, *n* (%)	365 (1.3)	
History of PCI, *n* (%)	389 (1.4)	
History of CABG, *n* (%)	692 (2.5)	
History of angina, *n* (%)	273 (1.0)	
History of PVD, *n* (%)	1035 (3.7)	
History of amputation, *n* (%)	1156 (4.2)	
History of hypoglycaemia, *n* (%)	3898 (14.0)	
History of hyperglycaemia, *n* (%)	3941 (14.2)	
History of ESRD, *n* (%)	154 (0.6)	

Values are presented as means±SD or *n* (%)

CABG, coronary artery bypass graft; CHF, congestive heart failure; ESRD, end-stage renal disease; MI, myocardial infarction; PCI, percutaneous coronary intervention; PVD, peripheral vascular disease

The lowest and highest levels of the continuous risk factors were selected to encompass the normal ranges of their values and contain mean values of the population of patients with type 1 diabetes used to develop the simulation model, referred to as the real-world population [27] (see Table 1). With these selected risk factors and their levels, the life expectancy table contained 1024 cells, each representing an individual with a unique combination of the baseline levels from six risk factors. To execute the simulation for each individual, the risk factors not included in the table, referred to as ‘hidden’ variables, were assigned values that represented the values of those risk factors in the real-world population (see Table 1). Based on this principle, we assigned mean values of the continuous risk factors in the real-world population to the corresponding hidden risk factors of any individual in the life expectancy table. However, this approach was not applicable for the binary risk factors, which could not take on values different from the levels used in the statistical model fitting (see Simulation model for type 1 diabetes, below, for details). For example, 14% of the real-world population had a history of hypoglycaemia (see Table 1); however, it was not possible to assign the value of 0.14 to the variable indicating the history of hypoglycaemia, as this variable could only receive a value of 0 or 1. Therefore, for each individual in the life expectancy table, we created a synthetic cohort containing 1000 individuals in which values of the hidden binary risk factors followed a joint distribution similar to that in the real-world population (see Creation of the synthetic cohorts, below, for details). In this way, mean life expectancy of a synthetic cohort represents the life expectancy of an ‘average’ individual with respect to the values of those binary risk factors. As a result, we created 1024 synthetic cohorts, each of which was unique in terms of the combined levels of six risk factors but identical in values of the other characteristics. In this way, any difference in life expectancy between individuals in the table would be attributed only to the differences in the levels of the risk factors included in the table. Then, we ran the simulations for all these cohorts to estimate the life expectancies of all cells in the life expectancy table (see Simulations and outcomes aggregation, below, for details).

### Simulation model for type 1 diabetes

The simulation model used in this study comprised 14 parametric proportional hazards equations to estimate the risk of type 1 diabetes-related complications and death, and ten equations to predict changes over time in risk factors of the patients [25]. These equations were developed using data from the real-world population selected from the Swedish National Diabetes Register [27]. To minimise the risk of including individuals with type 2 diabetes, only those who were younger than 30 years of age at diabetes diagnosis and had at least one prescription of insulin annually and no prescriptions for metformin were included in the study cohort. The exclusion of metformin prescriptions was due to the infrequent use of this medication in the treatment of patients with type 1 diabetes in Sweden. As a result, the population used for the model development contained 27,841 individuals with type 1 diabetes, recorded between 1 January 2002 and 31 December 2011, with a mean follow-up time of 7 years. Predictors in the equations for risk and risk progression were selected from 41 potential variables plus variables representing the interaction effects between patient characteristics and age. Except for BMI, all the categorical predictors were binary variables. These categorical variables were coded as dummy variables, with 0 indicating the reference and 1 indicating the level compared with the reference.

For the statistical analyses, all HbA_1c_ values were converted to standard levels according to the US National Glycohemoglobin Standardization Program [28]. Values of eGFR were calculated using the Chronic Kidney Disease Epidemiology Collaboration equation [29]. All equations for risk and risk factor progression were internally validated by comparing the predictions and observed data, with the results showing that there was a high agreement between the simulated and observed values (see our previous publication: Figure 2 in the main text and Figure S2 in the supplementary material [25]). A full description of the simulation model, including the identification of the events, the definitions of the risk factors, the equations and the validation exercises, can be found in our previous study [25]. Briefly, the model predicts occurrence of the following events: fatal and non-fatal myocardial infarction; fatal and non-fatal stroke; heart failure; peripheral vascular disease; hypoglycaemia; hyperglycaemia; amputation; end-stage renal disease; percutaneous coronary intervention; coronary artery bypass graft; and all-cause death. The model also simulates changes over time in the values of the following risk factors: HbA_1c_; BMI; systolic BP; triacylglycerols; HDL-cholesterol; LDL-cholesterol; eGFR; smoking status; microalbuminuria; and macroalbuminuria. The model was structured to capture the interdependencies between risk factor progression, histories of complications and future occurrence of complications and death, which were necessary for an unbiased estimation of life expectancy.

### Creation of the synthetic cohorts

As mentioned earlier, 1024 synthetic cohorts were created, each represented by an individual with a unique combination of the levels of the risk factors included in the life expectancy table. The dataset for each cohort was constructed to contain 1000 observations and all variables (i.e. predictors) required to execute the equations for risk and risk factor progression. Four categories of predictors were taken into consideration in the setting of their values: (1) risk factors included in the life expectancy table, which were assigned the pre-specified levels; (2) binary variables not included in the life expectancy table, which were assigned values sampled using the joint Bernoulli distributions of these variables in the real-world population; (3) continuous variables not included in the life expectancy table, which were assigned mean values of these variables in the real-world population; and (4) variables representing the interaction effects between age and the variables in the first three categories.

The binary variables included those indicating histories of complications, microalbuminuria and macroalbuminuria, and the number of prior complications (see Table 1 and ESM Table 1). Our previous study [25] showed that the occurrence of a complication influenced the subsequent occurrence of other complications; therefore, we sampled the histories of the complications using a joint distribution, and microalbuminuria and macroalbuminuria using another joint distribution to partly capture the interdependencies between them. The sampling of the values of a set of interdependent variables was carried out as follows. First, we identified every unique combination of the values of these variables in the real-world population and associated each unique combination with a unique identifier. Then, we assigned each observation in the real-world population with an identifier corresponding to the combined values of the variables in that observation. Next, we calculated the proportions of the population with different identifiers, which represented the probability mass function of the identifiers. Based on the probability mass functions, we constructed the cumulative distribution function of the identifiers, and used this to sample the identifiers based on the inverse transform method [30]. Finally, we assigned the variables in the synthetic cohort with the values corresponding to the sampled identifiers. After the continuous variables were assigned values and the binary variables were sampled, the variables representing the interaction effects between those variables and age were created and their values calculated to complete the set of predictors in each synthetic cohort.

### Simulations and outcomes aggregation

For each synthetic cohort, we ran the simulation until all individuals had died. During the simulation, the occurrence of the events and changes in risk factor values were predicted in annual cycles using the equations for risk and risk factor progression as described above. The remaining life expectancy of an individual was defined as time from the baseline age to death. Details of the simulation process can be found in our previous publication [25]. To reduce the first-order (stochastic) uncertainty, which relates to the fact that individuals with identical characteristics may experience different outcomes, we repeated the simulation for each cohort 100 times and calculated the life expectancy of each individual as mean across 100 replications. We then calculated mean life expectancy and its SD based on the life expectancies of 1000 individuals in each cohort, to represent the life expectancy and its variation for an average person in that cohort. Then, we created a table with 1024 cells, each of which displays the life expectancy corresponding to a unique combination of risk factor levels. The table was colourised to display a gradient of the outcomes within each age and sex stratum (considered as non-modifiable risk factors), in which green represents better outcomes and red worse.

We used the model developed in Stata 15.0 (StataCorp, College Station, TX, USA) to run the simulations, and R version 4.02 (R Foundation for Statistical Computing, Vienna, Austria) [31] to create the synthetic cohorts and compile the simulated outcomes.

## Results

**Fig. 1:**
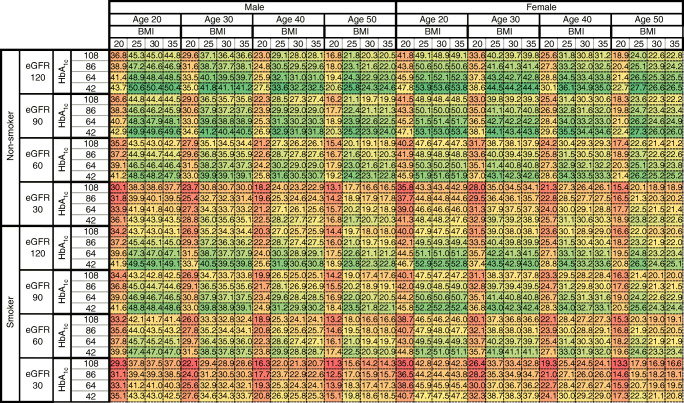
Life expectancy of individuals with type 1 diabetes and with specific baseline levels of sex, age (years), BMI (kg/m^2^), current smoking status, eGFR (ml min^−1^ [1.73 m]^−2^) and HbA_1c_ (mmol/mol). The colour gradient within each age–sex stratum goes from green (higher life expectancy) to dark red (lower life expectancy). Value in each cell represents the number of years from the baseline age to death

**Fig. 2:**
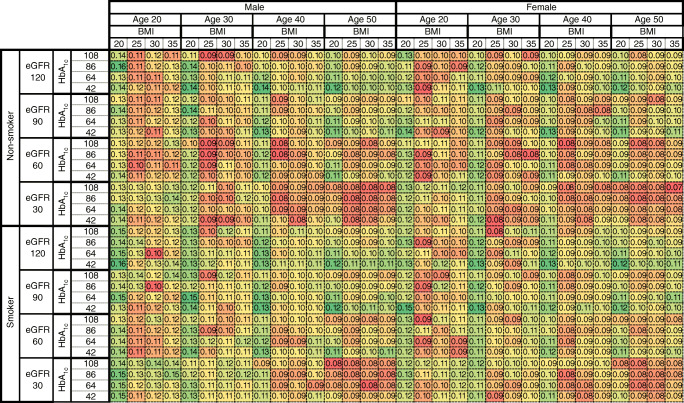
SEs of the life expectancies of individuals with type 1 diabetes and with specific baseline levels of sex, age (years), BMI (kg/m^2^), current smoking status, eGFR (ml min^−1^ [1.73 m]^−2^) and HbA_1c_ (mmol/mol). The colour gradient within each age–sex stratum goes from green (larger SEs) to dark red (smaller SEs). This figure complements Fig. 1

Figure 1 provides life expectancies of 1024 patients with specific risk factor levels and Fig. 2 provides the SEs of these life expectancies. There was a substantial variation in life expectancy across individuals with different risk factor levels. For example, life expectancy of 20-year-old men varied from 29.3 years to 50.6 years, constituting a gap of 21.3 years between those with worst and best risk factor levels. In 20-year-old women, this gap was 18.9 years (life expectancy range 35.0–53.9 years).

The variation in life expectancy was a function of the combination of risk factor values, with HbA_1c_ and eGFR consistently showing a negative and positive correlation, respectively, with life expectancy at any level combination of other risk factors. Individuals with the lowest level (20 kg/m^2^) and highest level of BMI (35 kg/m^2^) had a lower life expectancy compared with those with a BMI of 25 kg/m^2^. Non-smokers and women had a higher life expectancy than smokers and men, respectively, with the difference in life expectancy ranging from 0.4 years to 2.7 years between non-smokers and smokers, and from 1.9 years to 5.9 years between women and men, depending on levels of other risk factors.

Figure 1 can be used to estimate the benefits associated with the differences in lifestyle factors. For example, a male aged 30 years, with eGFR of 30 ml min^−1^ [1.73 m]^−2^, who was a smoker, and had a BMI of 35 kg/m^2^ and an HbA_1c_ of 108 mmol/mol (12%) would have a life expectancy of 28.6 years. However, if the HbA_1c_ of this individual was lowered to 42 mmol/mol (6%), his life expectancy would increase to 33.7 years; in addition to this HbA_1c_ lowering, if the eGFR was increased to 120 ml min^−1^ [1.73 m]^−2^, his life expectancy would further increase to 39.8 years. In addition to these improvements in HbA_1c_ and eGFR, if BMI were reduced to 25 kg/m^2^ then life expectancy would further increase to 40.5 years, and if the person were a non-smoker the life expectancy would be 41.8 years. The gain in life expectancy associated with the combined improvement of the above-mentioned risk factors would be 13.2 years in this instance.

Another version of the life expectancy table, using a greyscale-based gradient, is provided in ESM Fig. 1. Figures representing age at death are also provided in ESM Fig. 2 (colourised within each age and sex stratum) and ESM Fig. 3 (colourised across the whole population). An interactive web application for the life expectancy table is available at https://antranduy.shinyapps.io/le-t1d/. This application is best viewed on a full HD widescreen computer monitor.

## Discussion

We have generated an age- and sex-stratified life expectancy table that demonstrates the impact of different combinations of risk factor levels on life expectancy in individuals with type 1 diabetes. This adds to the existing literature by providing estimates of the impact of both modifiable and non-modifiable variables on the average life expectancy. Our results are presented in a similar format to other risk and life expectancy tables that are widely used [19, 32, 33], in which various combinations of risk factors organised by columns and rows permit specific life expectancies to be read from the table.

Using our table, the life expectancy of a 20-year-old man with type 1 diabetes was between 29.3 years and 50.6 years, and that of a 20-year-old woman between 35.0 years and 53.9 years. These life expectancies were substantially lower than the 59.4 and 63.3 years for 20-year-old men and women, respectively, derived from the Swedish general population in 2007 [34]. We found large variations in life expectancies of individuals with the same non-modifiable variables (i.e. sex or age), as well as the same levels of modifiable variables such as HbA_1c_, eGFR, BMI and smoking status. Changes in the levels of modifiable variables accounted for the broad range of possible life expectancies reported.

The life expectancy table we generated provides pertinent information on a patient’s prognosis, as well as potential life expectancy gains associated with improvement of important risk factors. This information is valuable to patients and clinicians in setting a goal for changing lifestyle and treatment. These results clearly demonstrate the potential for increasing life expectancy by improving levels of modifiable risk factors. This information may also be used in other fields (e.g. insurance companies could use our life expectancy table to calculate annuities for individuals with type 1 diabetes [35]). It should be noted that the estimated life expectancies are conditional on an individual surviving to the baseline age presented in the table. As a result, mean age at death (current age plus life expectancy) increased with increasing baseline age in individuals with identical levels of other risk factors (see ESM Fig. 2). This is because a younger individual would be at risk for a number of years before he/she reached the older baseline age. Therefore, one should not make the interpretation that an older patient is ‘healthier’ than a younger one with identical risk factors.

A substantial gap in life expectancy between individuals with type 1 diabetes and the general population has been reported on a number of occasions [1]. Recent reports [36] indicate that infectious diseases such as coronavirus disease-2019 (COVID-19) have a disproportionate impact on individuals with type 1 diabetes, with a potential 3.5-fold increase in risk of dying in hospital with COVID-19. Our risk factor stratified life expectancy table can support the estimation of the burden of type 1 diabetes, as well as the potential loss from emerging diseases such as COVID-19.

The life expectancy table developed in this study allows for assessment of the impact of six important risk factors. It is possible to expand this table to include more risk factors (e.g. lipid levels) and increase the number of levels for each continuous risk factor. However, the use of a table with several thousands of cells may not be practical. In this eventuality, the availability of an electronic life expectancy calculator would be extremely helpful, and development of such a calculator is an objective for our future work.

### Strengths and limitations

This is the first study that developed a life expectancy table for individuals with type 1 diabetes. The analysis was based on a comprehensive simulation model derived from a large type 1 diabetes registry population [25]. The data were collected prospectively and had a high level of coverage across the Swedish national diabetic population. The registry contains detailed information on clinical characteristics and laboratory measures which were used in this study for the development of the synthetic cohorts. The simulation model [25] has been internally validated, with the predicted event rates closely matching those observed in the real-world population.

Our study has limitations. The data used to develop the simulation model were recorded between 1 January 2002 and 31 December 2011. Therefore, the overall secular trend of reduced mortality and the type 1 diabetes-specific improvement in life expectancy in the period after 2011 was not captured in the model [25]. As a consequence, the life expectancies estimated in this study might be slightly lower than those of the current patients. It is therefore important to regularly update the equations in the simulation model using more recent data and to use the updated simulation model to re-estimate the life expectancies in the table.

Due to the observational nature of the registry data, the equations in the simulation model [25] describe associations and not necessarily a causal link between the predictors and the outcomes. Therefore, care should be taken when making causal assumptions about the impact of changes in risk factors on the life expectancies in the table. Although the simulation model used to estimate the life expectancies was internally validated, it has not been externally validated using datasets other than the Swedish National Diabetes Register [27]. Therefore, it is currently unclear whether the life expectancies we estimated also represent those of patients with type 1 diabetes in geographical regions other than Sweden.

### Conclusions

The life expectancy table generated in this study shows a substantial variation in life expectancy across individuals with different modifiable risk factors. This indicates a large scope for increasing life expectancy via optimisation of the risk factor levels. The table allows for rapid communications of risk in an easily understood format between healthcare professionals, health economists, researchers, policy makers and patients. Particularly, it supports clinicians in their discussion with the patients about benefits of improving risk factors in terms years of life gained, an important outcome measure to the patients.

## Supplementary Information

ESM (PDF 717 kb)

## Abbreviation

COVID-19: Coronavirus disease-2019
