# Supplementary material for: Development of a life expectancy table for individuals with type 1 diabetes
Source: Diabetologia. 2021 Jul 26;64(10):2228–36. doi: 10.1007/s00125-021-05503-6 (PMC8310903; doi:10.1007/s00125-021-05503-6)
Supplement: Supplementary file 1 — (PDF 717 kb) [file 125_2021_5503_MOESM1_ESM.pdf]

Development of a life expectancy table for individuals with type 1 diabetes

An Tran-Duy, Josh Knight, Philip M. Clarke, Ann-Marie Svensson, Björn Eliasson, Andrew J. Palmer

Electronic Supplementary Materials (ESM)

ESM Table 1.

Auxiliary variables required as input for the simulation model. Baseline line values were derived from the real-world population (*n* = 27,841)

Variables	Baseline values used for sampling*
Time since last myocardial infarction, years	3.1 (1.38)
Time since last stroke, years	2.7 (0.6)
Time since diagnosis of heart failure, years	2.8 (0.5)
Time since last percutaneous coronary intervention, years	3.2 (0.6)
Time since last coronary artery bypass graft, years	3.5 (1.7)
Time since diagnosis of angina, years	2.7 (0.5)
Time since diagnosis of peripheral vascular disease, years	4.1 (6.1)
Time since last amputation, years	4.5 (6.5)
Time since last hypoglycaemia, years	5.5 (2.1)
Time since last hyperglycaemia, years	5.2 (2.0)
Time since diagnosis of end-stage renal disease, years	2.9 (0.3)
Indicator of prior occurrence of exactly two events†, *n* (%)	1,760 (6.3)
Indicator of prior occurrence of exactly three events†, *n* (%)	369 (1.33)
Indicator of prior occurrence of exactly four events†, *n* (%)	82 (0.29)
Indicator of prior occurrence of exactly five events†, *n* (%)	14 (0.05)
Indicator of prior occurrence of six or more events†, *n* (%)	3 (0.01)

* Values represent means and standard deviations, except for those expressed as *n (%).*

† An event indicates a type 1 diabetes-related complication.

ESM Fig. 1.

Life expectancy of individuals with type 1 diabetes and with specific baseline levels of sex, age (years), body mass index (BMI; kg/m^2), current smoking status, estimated glomerular filtration rate (eGFR; mL min^{-1} [1.73 m]$^{-2}$), and HbA1c (mmol/mol). The colour gradient was generated on a grayscale within each age-sex stratum, with the lighter colour representing the higher life expectancy. Value in each cell represents the number of years from the baseline age to death

				Male															
				Age 20				Age 30				Age 40				Age 50			
				BMI				BMI				BMI				BMI			
				20	25	30	35	20	25	30	35	20	25	30	35	20	25	30	35
Non-smoker	eGFR 120	HbA1c	108	36.8	45.3	45.0	44.8	29.6	37.1	36.4	36.6	23.0	29.1	28.0	28.1	16.8	21.8	20.3	20.5
			86	38.9	47.2	46.6	46.9	31.6	38.7	37.7	38.1	24.8	30.5	29.5	29.6	18.0	23.1	21.6	22.0
			64	41.4	48.9	48.4	48.5	33.5	40.1	39.5	39.7	25.9	32.1	31.0	31.0	19.4	24.3	22.9	23.0
			42	43.7	50.6	50.4	50.4	35.0	41.8	41.1	41.2	27.5	33.6	32.2	32.5	20.6	25.8	24.3	24.6
	eGFR 90	HbA1c	108	36.6	44.8	44.4	44.5	29.0	36.5	35.7	35.8	22.3	28.5	27.3	27.4	16.2	21.1	19.7	19.9
			86	38.3	46.6	46.2	45.9	30.6	37.9	37.2	37.6	23.9	29.9	29.0	29.0	17.7	22.4	21.1	21.3
			64	40.7	48.3	47.9	48.1	33.0	39.6	38.8	38.9	25.3	31.3	30.2	30.3	18.9	23.9	22.6	22.5
			42	42.9	49.9	49.6	49.6	34.6	41.2	40.4	40.5	26.9	32.9	31.9	31.8	20.3	25.2	23.9	24.0
	eGFR 60	HbA1c	108	35.2	43.5	43.0	42.7	27.9	35.1	34.5	34.4	21.2	27.3	26.2	26.1	15.4	20.1	19.1	18.9
			86	37.2	44.9	44.7	44.4	29.6	36.8	35.9	35.9	22.6	28.7	27.8	27.6	16.7	21.6	20.1	20.3
			64	39.1	46.5	46.4	46.4	31.5	38.2	37.4	37.3	24.2	30.2	29.0	29.0	17.9	23.0	21.6	21.6
			42	41.2	48.5	48.2	47.9	33.0	39.9	39.1	39.1	25.8	31.6	30.5	30.7	19.2	24.2	23.1	22.8
	eGFR 30	HbA1c	108	30.1	38.3	38.6	37.7	23.7	30.8	30.7	30.0	18.2	24.0	23.2	22.9	13.1	17.7	16.6	16.5
			86	31.8	39.9	40.1	39.5	25.4	32.7	32.3	31.4	19.6	25.3	24.6	24.3	14.2	18.9	17.9	17.8
			64	33.9	41.9	41.8	40.9	27.3	34.3	33.7	33.2	21.2	27.1	26.1	25.6	15.7	20.2	19.2	18.9
			42	36.1	43.9	43.9	43.5	28.8	36.0	35.6	35.1	22.5	28.2	27.7	27.2	16.8	21.7	20.7	20.3
Smoker	eGFR 120	HbA1c	108	34.2	43.7	43.0	43.1	26.9	35.2	34.3	34.4	20.3	27.0	25.7	25.9	14.4	19.7	18.0	18.0
			86	37.2	45.4	45.1	45.0	29.3	37.2	36.3	36.2	22.2	28.7	27.4	27.5	16.0	21.0	19.7	19.6
			64	39.6	47.3	47.0	47.1	31.5	38.7	37.7	37.9	24.0	30.3	28.9	29.1	17.5	22.6	21.1	21.2
			42	41.9	49.5	49.1	49.1	33.7	40.5	39.5	39.8	25.6	31.9	30.6	30.8	18.9	23.8	22.3	22.8
	eGFR 90	HbA1c	108	34.4	43.2	42.8	42.5	26.9	34.7	33.7	33.8	19.9	26.5	25.0	25.1	14.2	19.0	17.4	17.6
			86	36.8	45.0	44.7	44.6	29.1	36.5	35.5	35.6	21.7	28.1	26.9	26.9	15.5	20.2	18.9	19.1
			64	39.0	46.9	46.7	46.5	30.8	37.9	37.1	37.5	23.4	29.6	28.4	28.5	16.9	22.0	20.7	20.5
			42	41.6	48.8	48.4	48.6	33.0	39.8	38.9	39.1	24.9	31.2	29.9	30.2	18.4	23.5	21.8	22.1
	eGFR 60	HbA1c	108	33.2	42.1	41.7	41.4	26.0	33.3	32.8	32.4	18.9	25.3	24.1	24.1	13.2	18.0	16.6	16.6
			86	35.6	44.0	43.5	43.2	27.8	35.2	34.4	34.1	20.8	26.9	25.6	25.7	14.6	19.5	18.0	18.0
			64	37.8	45.7	45.2	45.1	29.7	36.4	35.9	36.0	22.3	28.6	27.4	27.1	16.1	21.0	19.4	19.7
			42	39.9	47.4	47.0	47.0	31.5	38.5	37.8	37.5	23.8	29.9	28.8	28.7	17.4	22.5	20.9	20.9
	eGFR 30	HbA1c	108	29.3	37.8	37.5	37.0	22.1	29.4	28.9	28.6	16.3	22.0	21.3	20.7	11.3	15.6	14.2	14.3
			86	31.1	39.4	39.3	38.5	24.0	31.2	30.5	30.3	17.7	23.7	22.9	22.6	12.5	17.0	15.9	15.7
			64	33.1	41.2	41.0	40.3	25.6	32.9	32.4	32.1	19.3	25.3	24.3	24.1	13.9	18.3	17.4	17.3
			42	35.1	43.3	43.0	42.5	27.6	34.6	34.3	33.7	20.8	26.9	25.8	25.3	15.1	19.8	18.6	18.5

				Female															
				Age 20				Age 30				Age 40				Age 50			
				BMI				BMI				BMI				BMI			
				20	25	30	35	20	25	30	35	20	25	30	35	20	25	30	35
Non-smoker	eGFR 120	HbA1c	108	41.8	49.1	48.9	49.1	33.6	40.2	39.7	39.8	25.6	31.8	30.8	31.2	18.9	24.0	22.6	22.8
			86	43.8	50.6	50.5	50.6	35.2	41.6	41.3	41.4	27.3	33.2	32.0	32.5	20.4	25.1	23.9	24.2
			64	45.9	52.1	52.1	52.3	37.3	43.2	42.7	42.8	28.8	34.5	33.4	33.8	21.4	26.5	25.3	25.5
			42	47.8	53.9	53.6	53.8	38.6	44.5	44.2	44.4	30.1	36.1	34.9	35.0	22.7	27.7	26.6	26.5
	eGFR 90	HbA1c	108	41.5	48.9	48.4	48.5	33.0	39.8	39.2	39.3	25.4	31.4	30.3	30.6	18.3	23.6	22.3	22.2
			86	43.3	50.1	50.0	50.0	35.0	41.1	40.7	40.8	26.9	32.8	31.6	32.0	19.8	24.7	23.4	23.4
			64	45.2	51.5	51.4	51.7	36.5	42.7	42.2	42.2	28.4	34.0	33.2	33.0	21.0	26.2	24.6	24.9
			42	47.1	53.1	53.0	53.4	38.1	44.1	43.4	43.8	29.6	35.5	34.4	34.6	22.4	27.3	26.0	26.0
	eGFR 60	HbA1c	108	40.2	47.6	47.4	47.3	31.7	38.7	38.1	37.9	24.2	30.4	29.3	29.3	17.4	22.6	21.4	21.2
			86	41.9	48.9	48.9	48.8	33.6	40.0	39.4	39.5	25.8	31.5	30.5	30.8	18.9	23.7	22.6	22.6
			64	43.9	50.3	50.2	50.1	35.1	41.4	40.8	40.8	27.3	32.9	32.1	32.2	20.3	25.1	23.9	23.8
			42	45.9	51.9	51.6	51.9	37.0	43.1	42.3	42.6	28.6	34.3	33.5	33.3	21.4	26.2	25.2	25.2
	eGFR 30	HbA1c	108	35.8	43.3	43.4	42.9	28.0	35.0	34.5	34.1	21.3	27.3	26.4	26.1	15.4	20.1	18.9	18.9
			86	37.7	44.8	44.8	44.6	29.5	36.4	36.1	35.7	22.8	28.5	27.7	27.5	16.5	21.3	20.3	20.2
			64	39.0	46.6	46.6	46.0	31.3	37.9	37.5	37.3	24.0	30.0	29.1	28.8	17.7	22.5	21.5	21.4
			42	41.3	48.4	48.2	47.6	32.9	39.7	39.2	38.9	25.7	31.1	30.6	30.3	18.9	23.8	22.8	22.6
Smoker	eGFR 120	HbA1c	108	40.0	47.9	47.5	47.6	31.5	39.0	38.0	38.1	23.6	30.1	28.9	29.0	16.6	22.0	20.3	20.6
			86	42.1	49.5	49.3	49.4	33.5	40.4	39.8	39.9	25.4	31.5	30.4	30.4	18.2	23.2	21.9	22.0
			64	44.5	51.1	51.0	51.2	35.7	42.2	41.3	41.5	27.1	33.1	32.1	32.1	19.5	24.8	23.2	23.4
			42	46.7	52.9	52.5	52.8	37.4	43.5	42.9	43.0	28.8	34.5	33.2	33.6	20.8	26.3	24.6	25.1
	eGFR 90	HbA1c	108	40.1	47.5	47.2	47.2	31.1	38.3	37.7	37.8	23.3	29.5	28.2	28.4	16.3	21.4	20.1	20.0
			86	42.0	49.0	48.5	49.0	32.8	39.7	39.4	39.5	24.7	31.2	29.8	30.0	17.6	22.9	21.3	21.5
			64	44.2	50.6	50.6	50.7	35.1	41.4	40.8	40.8	26.7	32.5	31.3	31.6	19.0	24.2	22.6	22.9
			42	45.8	52.2	52.2	52.4	36.8	43.0	42.3	42.4	28.3	34.0	32.7	33.1	20.5	25.6	24.3	24.4
	eGFR 60	HbA1c	108	38.7	46.5	46.2	46.0	30.1	37.3	36.8	36.6	22.1	28.4	27.2	27.3	15.3	20.3	19.0	19.1
			86	40.7	47.9	48.0	47.7	32.1	38.8	38.0	38.1	23.9	30.0	28.8	29.1	16.8	21.9	20.5	20.5
			64	43.0	49.5	49.4	49.3	33.7	40.2	39.7	39.7	25.4	31.4	30.3	30.4	18.2	23.2	21.9	21.9
			42	44.8	51.2	51.0	51.1	35.7	41.9	41.1	41.1	27.1	33.0	31.9	32.0	19.6	24.6	23.3	23.4
	eGFR 30	HbA1c	108	35.0	42.8	42.9	42.3	26.4	33.7	33.4	32.8	19.3	25.4	24.5	24.1	13.3	17.9	16.9	16.6
			86	36.5	44.2	44.4	43.8	28.2	35.3	34.9	34.7	21.0	27.1	26.0	26.0	14.6	19.5	18.2	18.1
			64	38.6	45.9	45.9	45.4	30.0	37.0	36.7	36.2	22.7	28.4	27.6	27.4	15.9	20.7	19.6	19.5
			42	40.7	47.7	47.5	47.2	32.3	38.7	38.3	37.9	24.1	30.0	29.2	29.0	17.3	22.3	21.1	20.8

ESM Fig. 2.

Age at death (in years) of individuals with type 1 diabetes and with specific baseline levels of sex, age (years), body mass index (BMI; kg/m^2), current smoking status, estimated glomerular filtration rate (eGFR; mL min^{-1} [1.73 m]$^{-2}$), and HbA1c (mmol/mol). The colour gradient was generated within each age-sex stratum, which goes from green (higher age at death) to dark red (lower age at death)

				Male																Female															
				Age 20				Age 30				Age 40				Age 50				Age 20				Age 30				Age 40				Age 50			
				BMI				BMI				BMI				BMI				BMI				BMI				BMI				BMI			
				20	25	30	35	20	25	30	35	20	25	30	35	20	25	30	35	20	25	30	35	20	25	30	35	20	25	30	35	20	25	30	35
Non-smoker	eGFR 120	HbA1c	108	56.8	65.3	65.0	64.8	59.6	67.1	66.4	66.6	63.0	69.1	68.0	68.1	66.8	71.8	70.3	70.5	61.8	69.1	68.9	69.1	63.6	70.2	69.7	69.8	65.6	71.8	70.8	71.2	68.9	74.0	72.6	72.8
			86	58.9	67.2	66.6	66.9	61.6	68.7	67.7	68.1	64.8	70.5	69.5	69.6	68.0	73.1	71.6	72.0	63.8	70.6	70.5	70.6	65.2	71.6	71.3	71.4	67.3	73.2	72.0	72.5	70.4	75.1	73.9	74.2
			64	61.4	68.9	68.4	68.5	63.5	70.1	69.5	69.7	65.9	72.1	71.0	71.0	69.4	74.3	72.9	73.0	65.9	72.1	72.1	72.3	67.3	73.2	72.7	72.8	68.8	74.5	73.4	73.8	71.4	76.5	75.3	75.5
			42	63.7	70.6	70.4	70.4	65.0	71.8	71.1	71.2	67.5	73.6	72.2	72.5	70.6	75.8	74.3	74.6	67.8	73.9	73.6	73.8	68.6	74.5	74.2	74.4	70.1	76.1	74.9	75.0	72.7	77.7	76.6	76.5
	eGFR 90	HbA1c	108	56.6	64.8	64.4	64.5	59.0	66.5	65.7	65.8	62.3	68.5	67.3	67.4	66.2	71.1	69.7	69.9	61.5	68.9	68.4	68.5	63.0	69.8	69.2	69.3	65.4	71.4	70.3	70.6	68.3	73.6	72.3	72.2
			86	58.3	66.6	66.2	65.9	60.6	67.9	67.2	67.6	63.9	69.9	69.0	69.0	67.7	72.4	71.1	71.3	63.3	70.1	70.0	70.0	65.0	71.1	70.7	70.8	66.9	72.8	71.6	72.0	69.8	74.7	73.4	73.4
			64	60.7	68.3	67.9	68.1	63.0	69.6	68.8	68.9	65.3	71.3	70.2	70.3	68.9	73.9	72.6	72.5	65.2	71.5	71.4	71.7	66.5	72.7	72.2	72.2	68.4	74.0	73.2	73.0	71.0	76.2	74.6	74.9
			42	62.9	69.9	69.6	69.6	64.6	71.2	70.4	70.5	66.9	72.9	71.9	71.8	70.3	75.2	73.9	74.0	67.1	73.1	73.0	73.4	68.1	74.1	73.4	73.8	69.6	75.5	74.4	74.6	72.4	77.3	76.0	76.0
	eGFR 60	HbA1c	108	55.2	63.5	63.0	62.7	57.9	65.1	64.5	64.4	61.2	67.3	66.2	66.1	65.4	70.1	69.1	68.9	60.2	67.6	67.4	67.3	61.7	68.7	68.1	67.9	64.2	70.4	69.3	69.3	67.4	72.6	71.4	71.2
			86	57.2	64.9	64.7	64.4	59.6	66.8	65.9	65.9	62.6	68.7	67.8	67.6	66.7	71.6	70.1	70.3	61.9	68.9	68.9	68.8	63.6	70.0	69.4	69.5	65.8	71.5	70.5	70.8	68.9	73.7	72.6	72.6
			64	59.1	66.5	66.4	66.4	61.5	68.2	67.4	67.3	64.2	70.2	69.0	69.0	67.9	73.0	71.6	71.6	63.9	70.3	70.2	70.1	65.1	71.4	70.8	70.8	67.3	72.9	72.1	72.2	70.3	75.1	73.9	73.8
			42	61.2	68.5	68.2	67.9	63.0	69.9	69.1	69.1	65.8	71.6	70.5	70.7	69.2	74.2	73.1	72.8	65.9	71.9	71.6	71.9	67.0	73.1	72.3	72.6	68.6	74.3	73.5	73.3	71.4	76.2	75.2	75.2
	eGFR 30	HbA1c	108	50.1	58.3	58.6	57.7	53.7	60.8	60.7	60.0	58.2	64.0	63.2	62.9	63.1	67.7	66.6	66.5	55.8	63.3	63.4	62.9	58.0	65.0	64.5	64.1	61.3	67.3	66.4	66.1	65.4	70.1	68.9	68.9
			86	51.8	59.9	60.1	59.5	55.4	62.7	62.3	61.4	59.6	65.3	64.6	64.3	64.2	68.9	67.9	67.8	57.7	64.8	64.8	64.6	59.5	66.4	66.1	65.7	62.8	68.5	67.7	67.5	66.5	71.3	70.3	70.2
			64	53.9	61.9	61.8	60.9	57.3	64.3	63.7	63.2	61.2	67.1	66.1	65.6	65.7	70.2	69.2	68.9	59.0	66.6	66.6	66.0	61.3	67.9	67.5	67.3	64.0	70.0	69.1	68.8	67.7	72.5	71.5	71.4
			42	56.1	63.9	63.9	63.5	58.8	66.0	65.6	65.1	62.5	68.2	67.7	67.2	66.8	71.7	70.7	70.3	61.3	68.4	68.2	67.6	62.9	69.7	69.2	68.9	65.7	71.1	70.6	70.3	68.9	73.8	72.8	72.6
Smoker	eGFR 120	HbA1c	108	54.2	63.7	63.0	63.1	56.9	65.2	64.3	64.4	60.3	67.0	65.7	65.9	64.4	69.7	68.0	68.0	60.0	67.9	67.5	67.6	61.5	69.0	68.0	68.1	63.6	70.1	68.9	69.0	66.6	72.0	70.3	70.6
			86	57.2	65.4	65.1	65.0	59.3	67.2	66.3	66.2	62.2	68.7	67.4	67.5	66.0	71.0	69.7	69.6	62.1	69.5	69.3	69.4	63.5	70.4	69.8	69.9	65.4	71.5	70.4	70.4	68.2	73.2	71.9	72.0
			64	59.6	67.3	67.0	67.1	61.5	68.7	67.7	67.9	64.0	70.3	68.9	69.1	67.5	72.6	71.1	71.2	64.5	71.1	71.0	71.2	65.7	72.2	71.3	71.5	67.1	73.1	72.1	72.1	69.5	74.8	73.2	73.4
			42	61.9	69.5	69.1	69.1	63.7	70.5	69.5	69.8	65.6	71.9	70.6	70.8	68.9	73.8	72.3	72.8	66.7	72.9	72.5	72.8	67.4	73.5	72.9	73.0	68.8	74.5	73.2	73.6	70.8	76.3	74.6	75.1
	eGFR 90	HbA1c	108	54.4	63.2	62.8	62.5	56.9	64.7	63.7	63.8	59.9	66.5	65.0	65.1	64.2	69.0	67.4	67.6	60.1	67.5	67.2	67.2	61.1	68.3	67.7	67.8	63.3	69.5	68.2	68.4	66.3	71.4	70.1	70.0
			86	56.8	65.0	64.7	64.6	59.1	66.5	65.5	65.6	61.7	68.1	66.9	66.9	65.5	70.2	68.9	69.1	62.0	69.0	68.5	69.0	62.8	69.7	69.4	69.5	64.7	71.2	69.8	70.0	67.6	72.9	71.3	71.5
			64	59.0	66.9	66.7	66.5	60.8	67.9	67.1	67.5	63.4	69.6	68.4	68.5	66.9	72.0	70.7	70.5	64.2	70.6	70.6	70.7	65.1	71.4	70.8	70.8	66.7	72.5	71.3	71.6	69.0	74.2	72.6	72.9
			42	61.6	68.8	68.4	68.6	63.0	69.8	68.9	69.1	64.9	71.2	69.9	70.2	68.4	73.5	71.8	72.1	65.8	72.2	72.2	72.4	66.8	73.0	72.3	72.4	68.3	74.0	72.7	73.1	70.5	75.6	74.3	74.4
	eGFR 60	HbA1c	108	53.2	62.1	61.7	61.4	56.0	63.3	62.8	62.4	58.9	65.3	64.1	64.1	63.2	68.0	66.6	66.6	58.7	66.5	66.2	66.0	60.1	67.3	66.8	66.6	62.1	68.4	67.2	67.3	65.3	70.3	69.0	69.1
			86	55.6	64.0	63.5	63.2	57.8	65.2	64.4	64.1	60.8	66.9	65.6	65.7	64.6	69.5	68.0	68.0	60.7	67.9	68.0	67.7	62.1	68.8	68.0	68.1	63.9	70.0	68.8	69.1	66.8	71.9	70.5	70.5
			64	57.8	65.7	65.2	65.1	59.7	66.4	65.9	66.0	62.3	68.6	67.4	67.1	66.1	71.0	69.4	69.7	63.0	69.5	69.4	69.3	63.7	70.2	69.7	69.7	65.4	71.4	70.3	70.4	68.2	73.2	71.9	71.9
			42	59.9	67.4	67.0	67.0	61.5	68.5	67.8	67.5	63.8	69.9	68.8	68.7	67.4	72.5	70.9	70.9	64.8	71.2	71.0	71.1	65.7	71.9	71.1	71.1	67.1	73.0	71.9	72.0	69.6	74.6	73.3	73.4
	eGFR 30	HbA1c	108	49.3	57.8	57.5	57.0	52.1	59.4	58.9	58.6	56.3	62.0	61.3	60.7	61.3	65.6	64.2	64.3	55.0	62.8	62.9	62.3	56.4	63.7	63.4	62.8	59.3	65.4	64.5	64.1	63.3	67.9	66.9	66.6
			86	51.1	59.4	59.3	58.5	54.0	61.2	60.5	60.3	57.7	63.7	62.9	62.6	62.5	67.0	65.9	65.7	56.5	64.2	64.4	63.8	58.2	65.3	64.9	64.7	61.0	67.1	66.0	66.0	64.6	69.5	68.2	68.1
			64	53.1	61.2	61.0	60.3	55.6	62.9	62.4	62.1	59.3	65.3	64.3	64.1	63.9	68.3	67.4	67.3	58.6	65.9	65.9	65.4	60.0	67.0	66.7	66.2	62.7	68.4	67.6	67.4	65.9	70.7	69.6	69.5
			42	55.1	63.3	63.0	62.5	57.6	64.6	64.3	63.7	60.8	66.9	65.8	65.3	65.1	69.8	68.6	68.5	60.7	67.7	67.5	67.2	62.3	68.7	68.3	67.9	64.1	70.0	69.2	69.0	67.3	72.3	71.1	70.8

ESM Fig. 3.

Age at death (in years) of individuals with type 1 diabetes and with specific baseline levels of sex, age (years), body mass index (BMI; kg/m^2), current smoking status, estimated glomerular filtration rate (eGFR; mL min^{-1} [1.73 m]$^{-2}$), and HbA1c (mmol/mol). The colour gradient was generated across all cells, which goes from green (higher age at death) to dark red (lower age at death)

			Male															
			Age 20				Age 30				Age 40				Age 50			
			BMI				BMI				BMI				BMI			
			20	25	30	35	20	25	30	35	20	25	30	35	20	25	30	35
Non-smoker	eGFR 120	HbA1c 108	56.8	65.3	65.0	64.8	59.6	67.1	66.4	66.6	63.0	69.1	68.0	68.1	66.8	71.8	70.3	70.5
Non-smoker	eGFR 120	HbA1c 86	58.9	67.2	66.6	66.9	61.6	68.7	67.7	68.1	64.8	70.5	69.5	69.6	68.0	73.1	71.6	72.0
Non-smoker	eGFR 120	HbA1c 64	61.4	68.9	68.4	68.5	63.5	70.1	69.5	69.7	65.9	72.1	71.0	71.0	69.4	74.3	72.9	73.0
Non-smoker	eGFR 120	HbA1c 42	63.7	70.6	70.4	70.4	65.0	71.8	71.1	71.2	67.5	73.6	72.2	72.5	70.6	75.8	74.3	74.6
Non-smoker	eGFR 90	HbA1c 108	56.6	64.8	64.4	64.5	59.0	66.5	65.7	65.8	62.3	68.5	67.3	67.4	66.2	71.1	69.7	69.9
Non-smoker	eGFR 90	HbA1c 86	58.3	66.6	66.2	65.9	60.6	67.9	67.2	67.6	63.9	69.9	69.0	69.0	67.7	72.4	71.1	71.3
Non-smoker	eGFR 90	HbA1c 64	60.7	68.3	67.9	68.1	63.0	69.6	68.8	68.9	65.3	71.3	70.2	70.3	68.9	73.9	72.6	72.5
Non-smoker	eGFR 90	HbA1c 42	62.9	69.9	69.6	69.6	64.6	71.2	70.4	70.5	66.9	72.9	71.9	71.8	70.3	75.2	73.9	74.0
Non-smoker	eGFR 60	HbA1c 108	55.2	63.5	63.0	62.7	57.9	65.1	64.5	64.4	61.2	67.3	66.2	66.1	65.4	70.1	69.1	68.9
Non-smoker	eGFR 60	HbA1c 86	57.2	64.9	64.7	64.4	59.6	66.8	65.9	65.9	62.6	68.7	67.8	67.6	66.7	71.6	70.1	70.3
Non-smoker	eGFR 60	HbA1c 64	59.1	66.5	66.4	66.4	61.5	68.2	67.4	67.3	64.2	70.2	69.0	69.0	67.9	73.0	71.6	71.6
Non-smoker	eGFR 60	HbA1c 42	61.2	68.5	68.2	67.9	63.0	69.9	69.1	69.1	65.8	71.6	70.5	70.7	69.2	74.2	73.1	72.8
Non-smoker	eGFR 30	HbA1c 108	50.1	58.3	58.6	57.7	53.7	60.8	60.7	60.0	58.2	64.0	63.2	62.9	63.1	67.7	66.6	66.5
Non-smoker	eGFR 30	HbA1c 86	51.8	59.9	60.1	59.5	55.4	62.7	62.3	61.4	59.6	65.3	64.6	64.3	64.2	68.9	67.9	67.8
Non-smoker	eGFR 30	HbA1c 64	53.9	61.9	61.8	60.9	57.3	64.3	63.7	63.2	61.2	67.1	66.1	65.6	65.7	70.2	69.2	68.9
Non-smoker	eGFR 30	HbA1c 42	56.1	63.9	63.9	63.5	58.8	66.0	65.6	65.1	62.5	68.2	67.7	67.2	66.8	71.7	70.7	70.3
Smoker	eGFR 120	HbA1c 108	54.2	63.7	63.0	63.1	56.9	65.2	64.3	64.4	60.3	67.0	65.7	65.9	64.4	69.7	68.0	68.0
Smoker	eGFR 120	HbA1c 86	57.2	65.4	65.1	65.0	59.3	67.2	66.3	66.2	62.2	68.7	67.4	67.5	66.0	71.0	69.7	69.6
Smoker	eGFR 120	HbA1c 64	59.6	67.3	67.0	67.1	61.5	68.7	67.7	67.9	64.0	70.3	68.9	69.1	67.5	72.6	71.1	71.2
Smoker	eGFR 120	HbA1c 42	61.9	69.5	69.1	69.1	63.7	70.5	69.5	69.8	65.6	71.9	70.6	70.8	68.9	73.8	72.3	72.8
Smoker	eGFR 90	HbA1c 108	54.4	63.2	62.8	62.5	56.9	64.7	63.7	63.8	59.9	66.5	65.0	65.1	64.2	69.0	67.4	67.6
Smoker	eGFR 90	HbA1c 86	56.8	65.0	64.7	64.6	59.1	66.5	65.5	65.6	61.7	68.1	66.9	66.9	65.5	70.2	68.9	69.1
Smoker	eGFR 90	HbA1c 64	59.0	66.9	66.7	66.5	60.8	67.9	67.1	67.5	63.4	69.6	68.4	68.5	66.9	72.0	70.7	70.5
Smoker	eGFR 90	HbA1c 42	61.6	68.8	68.4	68.6	63.0	69.8	68.9	69.1	64.9	71.2	69.9	70.2	68.4	73.5	71.8	72.1
Smoker	eGFR 60	HbA1c 108	53.2	62.1	61.7	61.4	56.0	63.3	62.8	62.4	58.9	65.3	64.1	64.1	63.2	68.0	66.6	66.6
Smoker	eGFR 60	HbA1c 86	55.6	64.0	63.5	63.2	57.8	65.2	64.4	64.1	60.8	66.9	65.6	65.7	64.6	69.5	68.0	68.0
Smoker	eGFR 60	HbA1c 64	57.8	65.7	65.2	65.1	59.7	66.4	65.9	66.0	62.3	68.6	67.4	67.1	66.1	71.0	69.4	69.7
Smoker	eGFR 60	HbA1c 42	59.9	67.4	67.0	67.0	61.5	68.5	67.8	67.5	63.8	69.9	68.8	68.7	67.4	72.5	70.9	70.9
Smoker	eGFR 30	HbA1c 108	49.3	57.8	57.5	57.0	52.1	59.4	58.9	58.6	56.3	62.0	61.3	60.7	61.3	65.6	64.2	64.3
Smoker	eGFR 30	HbA1c 86	51.1	59.4	59.3	58.5	54.0	61.2	60.5	60.3	57.7	63.7	62.9	62.6	62.5	67.0	65.9	65.7
Smoker	eGFR 30	HbA1c 64	53.1	61.2	61.0	60.3	55.6	62.9	62.4	62.1	59.3	65.3	64.3	64.1	63.9	68.3	67.4	67.3
Smoker	eGFR 30	HbA1c 42	55.1	63.3	63.0	62.5	57.6	64.6	64.3	63.7	60.8	66.9	65.8	65.3	65.1	69.8	68.6	68.5

			Female															
			Age 20				Age 30				Age 40				Age 50			
			BMI				BMI				BMI				BMI			
			20	25	30	35	20	25	30	35	20	25	30	35	20	25	30	35
Non-smoker	eGFR 120	HbA1c 108	61.8	69.1	68.9	69.1	63.6	70.2	69.7	69.8	65.6	71.8	70.8	71.2	68.9	74.0	72.6	72.8
Non-smoker	eGFR 120	HbA1c 86	63.8	70.6	70.5	70.6	65.2	71.6	71.3	71.4	67.3	73.2	72.0	72.5	70.4	75.1	73.9	74.2
Non-smoker	eGFR 120	HbA1c 64	65.9	72.1	72.1	72.3	67.3	73.2	72.7	72.8	68.8	74.5	73.4	73.8	71.4	76.5	75.3	75.5
Non-smoker	eGFR 120	HbA1c 42	67.8	73.9	73.6	73.8	68.6	74.5	74.2	74.4	70.1	76.1	74.9	75.0	72.7	77.7	76.6	76.5
Non-smoker	eGFR 90	HbA1c 108	61.5	68.9	68.4	68.5	63.0	69.8	69.2	69.3	65.4	71.4	70.3	70.6	68.3	73.6	72.3	72.2
Non-smoker	eGFR 90	HbA1c 86	63.3	70.1	70.0	70.0	65.0	71.1	70.7	70.8	66.9	72.8	71.6	72.0	69.8	74.7	73.4	73.4
Non-smoker	eGFR 90	HbA1c 64	65.2	71.5	71.4	71.7	66.5	72.7	72.2	72.2	68.4	74.0	73.2	73.0	71.0	76.2	74.6	74.9
Non-smoker	eGFR 90	HbA1c 42	67.1	73.1	73.0	73.4	68.1	74.1	73.4	73.8	69.6	75.5	74.4	74.6	72.4	77.3	76.0	76.0
Non-smoker	eGFR 60	HbA1c 108	60.2	67.6	67.4	67.3	61.7	68.7	68.1	67.9	64.2	70.4	69.3	69.3	67.4	72.6	71.4	71.2
Non-smoker	eGFR 60	HbA1c 86	61.9	68.9	68.9	68.8	63.6	70.0	69.4	69.5	65.8	71.5	70.5	70.8	68.9	73.7	72.6	72.6
Non-smoker	eGFR 60	HbA1c 64	63.9	70.3	70.2	70.1	65.1	71.4	70.8	70.8	67.3	72.9	72.1	72.2	70.3	75.1	73.9	73.8
Non-smoker	eGFR 60	HbA1c 42	65.9	71.9	71.6	71.9	67.0	73.1	72.3	72.6	68.6	74.3	73.5	73.3	71.4	76.2	75.2	75.2
Non-smoker	eGFR 30	HbA1c 108	55.8	63.3	63.4	62.9	58.0	65.0	64.5	64.1	61.3	67.3	66.4	66.1	65.4	70.1	68.9	68.9
Non-smoker	eGFR 30	HbA1c 86	57.7	64.8	64.8	64.6	59.5	66.4	66.1	65.7	62.8	68.5	67.7	67.5	66.5	71.3	70.3	70.2
Non-smoker	eGFR 30	HbA1c 64	59.0	66.6	66.6	66.0	61.3	67.9	67.5	67.3	64.0	70.0	69.1	68.8	67.7	72.5	71.5	71.4
Non-smoker	eGFR 30	HbA1c 42	61.3	68.4	68.2	67.6	62.9	69.7	69.2	68.9	65.7	71.1	70.6	70.3	68.9	73.8	72.8	72.6
Smoker	eGFR 120	HbA1c 108	60.0	67.9	67.5	67.6	61.5	69.0	68.0	68.1	63.6	70.1	68.9	69.0	66.6	72.0	70.3	70.6
Smoker	eGFR 120	HbA1c 86	62.1	69.5	69.3	69.4	63.5	70.4	69.8	69.9	65.4	71.5	70.4	70.4	68.2	73.2	71.9	72.0
Smoker	eGFR 120	HbA1c 64	64.5	71.1	71.0	71.2	65.7	72.2	71.3	71.5	67.1	73.1	72.1	72.1	69.5	74.8	73.2	73.4
Smoker	eGFR 120	HbA1c 42	66.7	72.9	72.5	72.8	67.4	73.5	72.9	73.0	68.8	74.5	73.2	73.6	70.8	76.3	74.6	75.1
Smoker	eGFR 90	HbA1c 108	60.1	67.5	67.2	67.2	61.1	68.3	67.7	67.8	63.3	69.5	68.2	68.4	66.3	71.4	70.1	70.0
Smoker	eGFR 90	HbA1c 86	62.0	69.0	68.5	69.0	62.8	69.7	69.4	69.5	64.7	71.2	69.8	70.0	67.6	72.9	71.3	71.5
Smoker	eGFR 90	HbA1c 64	64.2	70.6	70.6	70.7	65.1	71.4	70.8	70.8	66.7	72.5	71.3	71.6	69.0	74.2	72.6	72.9
Smoker	eGFR 90	HbA1c 42	65.8	72.2	72.2	72.4	66.8	73.0	72.3	72.4	68.3	74.0	72.7	73.1	70.5	75.6	74.3	74.4
Smoker	eGFR 60	HbA1c 108	58.7	66.5	66.2	66.0	60.1	67.3	66.8	66.6	62.1	68.4	67.2	67.3	65.3	70.3	69.0	69.1
Smoker	eGFR 60	HbA1c 86	60.7	67.9	68.0	67.7	62.1	68.8	68.0	68.1	63.9	70.0	68.8	69.1	66.8	71.9	70.5	70.5
Smoker	eGFR 60	HbA1c 64	63.0	69.5	69.4	69.3	63.7	70.2	69.7	69.7	65.4	71.4	70.3	70.4	68.2	73.2	71.9	71.9
Smoker	eGFR 60	HbA1c 42	64.8	71.2	71.0	71.1	65.7	71.9	71.1	71.1	67.1	73.0	71.9	72.0	69.6	74.6	73.3	73.4
Smoker	eGFR 30	HbA1c 108	55.0	62.8	62.9	62.3	56.4	63.7	63.4	62.8	59.3	65.4	64.5	64.1	63.3	67.9	66.9	66.6
Smoker	eGFR 30	HbA1c 86	56.5	64.2	64.4	63.8	58.2	65.3	64.9	64.7	61.0	67.1	66.0	66.0	64.6	69.5	68.2	68.1
Smoker	eGFR 30	HbA1c 64	58.6	65.9	65.9	65.4	60.0	67.0	66.7	66.2	62.7	68.4	67.6	67.4	65.9	70.7	69.6	69.5
Smoker	eGFR 30	HbA1c 42	60.7	67.7	67.5	67.2	62.3	68.7	68.3	67.9	64.1	70.0	69.2	69.0	67.3	72.3	71.1	70.8
